# A putative causal relationship between genetically determined female body shape and posttraumatic stress disorder

**DOI:** 10.1186/s13073-017-0491-4

**Published:** 2017-11-27

**Authors:** Renato Polimanti, Ananda B. Amstadter, Murray B. Stein, Lynn M. Almli, Dewleen G. Baker, Laura J. Bierut, Bekh Bradley, Lindsay A. Farrer, Eric O. Johnson, Anthony King, Henry R. Kranzler, Adam X. Maihofer, John P. Rice, Andrea L. Roberts, Nancy L. Saccone, Hongyu Zhao, Israel Liberzon, Kerry J. Ressler, Caroline M. Nievergelt, Karestan C. Koenen, Joel Gelernter, for The Psychiatric Genomics Consortium Posttraumatic Stress Disorder Workgroup, Lynn M. Almli, Dewleen G. Baker, Laura J. Bierut, Bekh Bradley, Lindsay A. Farrer, Joel Gelernter, Eric O. Johnson, Anthony King, Karestan C. Koenen, Henry R. Kranzler, Israel Liberzon, Adam X. Maihofer, Caroline M. Nievergelt, Kerry J. Ressler, John P. Rice, Andrea L. Roberts, Nancy L. Saccone, Hongyu Zhao

**Affiliations:** 1 Department of Psychiatry, Yale University School of Medicine and VA CT Healthcare Center, 116A2, 950 Campbell Avenue, West Haven, CT 06516 USA; 2 Department of Psychiatry, Virginia Commonwealth University, Richmond, VA USA; 3 Department of Psychiatry, University of California San Diego, La Jolla, CA USA; 4 Department of Family Medicine and Public Health, University of California San Diego, La Jolla, CA USA; 5 Veterans Affairs San Diego Healthcare System and Veterans Affairs Center of Excellence for Stress and Mental Health, La Jolla, CA USA; 6 Department of Psychiatry and Behavioral Sciences, Emory University, Atlanta, GA USA; 7 Department of Psychiatry, Washington University School of Medicine, St. Louis, MO USA; 8 Atlanta VA Medical Center, Atlanta, GA USA; 9 Department of Medicine, Biomedical Genetics Division, Boston University School of Medicine, Boston, MA USA; 10 Fellow Program and Behavioral Health and Criminal Justice Division RTI International, Research Triangle Park, NC USA; 11 Department of Psychiatry, University of Michigan, Ann Arbor, MI USA; 12 Department of Psychiatry, University of Pennsylvania Perelman School of Medicine and VISN 4 MIRECC, Crescenz VAMC, Philadelphia, PA USA; 13 Department of Environmental Health, Harvard T. H. Chan School of Public Health, Boston, MA USA; 14 Department of Genetics, Washington University School of Medicine, St. Louis, MO USA; 15 Department of Biostatistics, Yale University, New Haven, CT USA; 16 VA Ann Arbor Health System, Ann Arbor, MI USA; 17 Department of Psychiatry, Harvard University, Cambridge, MA USA; 18 Department of Psychiatry, McLean Hospital, Belmont, MA USA; 19 Department of Epidemiology, Harvard TH Chan School of Public Health, Boston, MA USA; 20 Psychiatric and Neurodevelopmental Genetics Unit, Center for Human Genetic Research, and Department of Psychiatry, Massachusetts General Hospital, Boston, MA USA; 21 Broad Institute of MIT and Harvard, Stanley Center for Psychiatric Research, Boston, MA USA; 22 Departments of Neuroscience and of Genetics, Yale University School of Medicine, New Haven, CT USA

**Keywords:** Trauma, Genetics, Women, Anthropometric traits

## Abstract

**Background:**

The nature and underlying mechanisms of the observed increased vulnerability to posttraumatic stress disorder (PTSD) in women are unclear.

**Methods:**

We investigated the genetic overlap of PTSD with anthropometric traits and reproductive behaviors and functions in women. The analysis was conducted using female-specific summary statistics from large genome-wide association studies (GWAS) and a cohort of 3577 European American women (966 PTSD cases and 2611 trauma-exposed controls). We applied a high-resolution polygenic score approach and Mendelian randomization analysis to investigate genetic correlations and causal relationships.

**Results:**

We observed an inverse association of PTSD with genetically determined anthropometric traits related to body shape, independent of body mass index (BMI). The top association was related to BMI-adjusted waist circumference (WC_adj_; R = –0.079, *P* < 0.001, Q = 0.011). We estimated a relative decrease of 64.6% (95% confidence interval = 27.5–82.7) in the risk of PTSD per 1-SD increase in WC_adj_. MR-Egger regression intercept analysis showed no evidence of pleiotropic effects in this association (P_pleiotropy_ = 0.979). We also observed associations of genetically determined WC_adj_ with age at first sexual intercourse and number of sexual partners (*P* = 0.013 and *P* < 0.001, respectively).

**Conclusions:**

There is a putative causal relationship between genetically determined female body shape and PTSD, which could be mediated by evolutionary mechanisms involved in human sexual behaviors.

**Electronic supplementary material:**

The online version of this article (doi:10.1186/s13073-017-0491-4) contains supplementary material, which is available to authorized users.

## Background

Posttraumatic stress disorder (PTSD) is a mental illness that affects susceptible individuals who have experienced, witnessed, or been confronted with an event involving actual or threatened dangers [1]. Individuals affected by PTSD present symptoms that, according to the DSM-5, include four diagnostic clusters (re-experiencing, avoidance, negative cognitions and mood, and arousal) [2]. At least one in nine American women meet lifetime diagnostic criteria for PTSD and women’s risk of PTSD is twice that of men [3]. Among women, PTSD is also more likely to be chronic and associated with greater functional impairment and distinct neurobiological profiles [4, 5]. Evidence suggests that women are more vulnerable than men to develop PTSD when exposed to similar traumas [6].

Women’s higher risk for PTSD likely arises from biologic and social/environmental factors. For example, sex differences in trauma exposure may partially explain the differential risk for PTSD. Women are more likely to be exposed to sexual violence, which has a high conditional risk of PTSD [7]. Alternatively, the pattern of results from twin studies suggests that PTSD may be more heritable in women than in men [8, 9] and results from a recent genome-wide investigation also demonstrate higher molecular heritability in women [10]. Sex hormones, particularly estradiol and progesterone, have been implicated as mediators of this risk, suggesting that their effects on biological, behavioral, and cognitive pathways augment the vulnerability to PTSD [11]. A recent epigenome-wide association study supported this possible mechanism; it reported an estrogen-dependent association of *HDAC4* with fear in female mice and women with PTSD [12].

Genetic data can be used to investigate this complex network of multiple factors. Polygenic risk score (PRS) analyses and Mendelian randomization (MR) studies are less biased by confounders than observational studies and can be used to investigate the predisposition to complex traits, identifying shared molecular pathways, pleiotropy, and causal relationships [13, 14]. Here, we used summary statistics from female-specific genome-wide association studies (GWAS) of traits that may be related to disease pathogenesis—i.e. anthropometric characteristics [15], reproductive physiology and behaviors [16], and educational attainment [17]—to examine the mechanisms underlying women’s vulnerability to PTSD. Although these traits were previously indicated as related to PTSD in women, testing a wide range of genetic correlations can provide useful etiological insights and help prioritize likely causal relationships [18]. A greater understanding of the biological and external social processes involved in PTSD will help to address the critically important interplay between two important public health issues: PTSD and women’s health.

## Methods

### Cohorts from the Psychiatric Genomics Consortium PTSD Workgroup

The individual data from the subjects included were made available by the members of the Psychiatric Genomics Consortium PTSD Workgroup. Details regarding participants, genotyping, quality control, imputation, and ancestry assignment were reported previously [10]. In the present study, we used data regarding female participants from seven cohorts: Collaborative Genetic Study of Nicotine Dependence [19]; Family Study of Cocaine Dependence [20]; Yale-Penn [21]; Grady Trauma Project [22]; Marine Resilience Study [23]; Nurses’ Health Study [24]; and Ohio National Guard [25]. Because the individuals in the GWAS that were used as sources for summary statistics for the traits of interest were of European descent, we restricted our analysis to women of that population. Quality control criteria for ancestry assignment and sex check are reported in our previous publication [10]. We considered imputed genotypes to maximize a consistent SNP (Single Nucleotide Polymorphism) panel between the training and testing sets. Imputed SNPs with high imputation quality (genotype call probability ≥ 0.8), minor allele frequency ≥ 1%, missingness per marker ≤ 5%, missingness per individual ≤ 5%, and Hardy-Weinberg equilibrium *P* > 10^–4^ were retained. After applying these quality control criteria, we retained information on 4,875,110 variants in a final sample of 3577 women (966 PTSD cases and 2611 trauma-exposed controls). To investigate the specificity of our findings for PTSD in women, we investigated a sample of 4628 men (501 PTSD cases and 4127 trauma-exposed controls) from the same cohorts of the Psychiatric Genomics Consortium PTSD Workgroup. The same quality control criteria were applied to the data from men. Principal component analysis of the final sample was conducted using PLINK 1.9 [26] after linkage disequilibrium (LD) pruning (R^2^ < 0.2).

### Polygenic risk score analysis

We conducted cross-phenotype PRS analyses using PRSice software [27] (available at http://prsice.info/). This method permits testing whether the polygenic components of inheritance have a substantial effect on multiple complex traits [28]. For polygenic profile scoring, we used summary statistics generated from female-specific GWAS of 17 traits (Table 1) conducted by the Genetic Investigation of ANthropometric Traits (GIANT) Consortium [29–32], the Reproductive Genetics (Reprogen) Consortium [33, 34], and the Social Science Genetic Association Consortium (SSGAC) [35, 36]. None of the PGC-PTSD cohorts was included in these previous GWAS and, thereby, no large overlap is expected among these samples. We considered multiple association *P* value thresholds (PT = 10^–8^, 10^–7^, 10^–6^, 10^–5^, 10^–4^, 0.001, 0.05, 0.3, 0.5) for SNP inclusion, i.e. we conducted a high-resolution analysis. The PRS were calculated after using *P* value-informed clumping with a LD cut-off of R^2^ = 0.3 within a 500-kb window, and excluding the Major Histocompatibility Complex region of the genome because of its complex LD structure. The PRS that were generated were fitted in regression models with adjustments for the top ten ancestry principal components to calculate Nagelkerke’s R as the figure of merit for prediction ability. We applied false discovery rate (FDR) multiple testing correction (Q < 0.05) to correct for the number of PRS and thresholds tested (a total of 170 tests). A gene ontology (GO) enrichment analysis was conducted based on the PRS results: the variants included in the significant PRS with direction concordant with PRS direction were considered in the enrichment analysis. Variants were mapped to the nearest genes and then the gene loci were entered into the enrichment analysis conducted using the PANTHER v11.1 Overrepresentation Test (release 20160715; Reference List: Homo Sapiens) [37]. FDR correction was applied to the enrichment results to account for multiple testing. The GO enrichment results were further investigated using REVIGO [38]. Specifically, GO enrichments were used to make a graph-based visualization considering an allowed similarity of 0.7, UniProt as reference database, and SimRel method as the semantic similarity measure.

**Table 1:** GWAS summary statistics used for polygenic risk scoring

Consortium	Trait	Abbreviation	Women N	Variant N	Data link
Genetic Investigation of ANthropometric Traits (GIANT)	Body mass index	BMI	128,698	2,494,584	http://portals.broadinstitute.org/collaboration/giant/index.php/GIANT_consortium_data_files
	BMI (age ≤ 50 years)	BMI_<50_	52,270	2,407,668	
	BMI (age ≥ 50 years)	BMI_>50_	86,731	2,407,629	
	Height	Height	64,811	2,748,519	
	Hip circumference	HIP	85,973	2,466,814	
	HIP adjusted for BMI	HIP_adj_	84,787	2,466,814	
	Waist circumference	WC	90,543	2,444,344	
	WC adjusted for BMI	WC_adj_	89,759	2,473,027	
	Waist–hip ratio	WHR	85,466	2,466,093	
	WHR adjusted for BMI	WHR_adj_	84,222	2,467,768	
	WHRadj (age ≤ 50 years)	WHR_adj<50_	34,523	2,366,527	
	WHRadj (age ≥ 50 years)	WHR_adj>50_	56,150	2,366,567	
Reproductive Genetics (Reprogen)	Age at menarche	Menarche	182,416	2,441,881	http://www.reprogen.org/data_download.html
	Age at menopause	Menopause	69,360	2,418,695	
Social Science Genetic Association Consortium (SSGAC)	Age at first birth	AFB	158,856	2,470,136	http://www.thessgac.org/data
	Education years	EDU	122,427	7,643,300	
	Number of children ever born	NEB	222,837	2,471,862	

### Two-sample Mendelian randomization analysis

The two-sample MR analysis was conducted using the R package *MendelianRandomization* (available at https://cran.r-project.org/web/packages/MendelianRandomization/index.html) and the GWAS summary statistics. This is a strategy to test cause–effect associations, in which evidence on the associations of genetic variants (i.e. instrumental variable) with the risk factor (i.e. exposure) and with the outcome comes from two sources [39]. We used SNPs associated with waist circumference adjusted for body mass index (BMI) (WC_adj_ P < 10^-8^ *N* = 31), which showed the most significant correlation with PTSD in our PRS analysis (FDR Q = 0.011), as the instrumental variable. The SNP-exposure and SNP-outcome coefficients (WC_adj_ and PTSD associations, respectively) were combined using an inverse-variance-weighted approach to give an overall estimate of the causal effect. To verify the stability of the results, we compared the findings obtained from inverse-variance-weighted analysis to the results of other MR approaches, including simple median, weighted median, and MR-Egger regression. Different methods have sensitivities to different potential issues, accommodate different scenarios, and vary in their statistical efficiency. Comparing the results of different methods permits validation of the analyses. MR-Egger regression intercept was considered to test for the presence of pleiotropic effects of the SNPs on the outcome [40]. Due to the lack of genome-wide significant loci for PTSD, it was not possible to apply reverse MR to test the effect of PTSD-associated alleles on WC_adj_ to confirm the direction of the causal relationship between these traits [41]. However, we used a reverse PRS analysis of PTSD to predict WC_adj_ and other anthropometric traits based on GWAS summary statistics. This reverse PRS analysis was conducted using PRSice software [27]. To follow up a possible “evolutionary link” between female body shape and PTSD, we tested the association of genetically determined WC_adj_ with age at first sexual intercourse (AFS) and number of sexual partners (NSP). For AFS, we used female-specific summary statistics from a recent GWAS, including women of European descent only [42]. This previous GWAS was conducted considering a range of AFS, which also includes a small fraction of individuals who had their first sexual intercourse before 12 years of age. For NSP, we used UK Biobank data on a total of 58,356 unrelated women of European descent. We calculated the association of the top 31 SNPs associated with WC_adj_ (*P* < 10^–8^) with NSP using a linear regression model. Before being entered into the analysis, NSP was adjusted for age and the top ten ancestry principal components and then normalized using appropriate Box–Cox power transformations.

### Data availability

Data supporting the findings of this study are available within the article and its Additional files. GWAS Summary level data used to calculate PRS in this study were obtained from links reported in Table 1.

## Results

We used summary statistics generated from female-specific GWAS of 17 traits (Table 1) to yield polygenic profile scoring and tested the association of the derived PRS with PTSD considering multiple PT and using samples from the Psychiatric Genomics Consortium PTSD Workgroup (966 PTSD cases and 2611 trauma-exposed controls). Six PRS showed correlations with PTSD that survived FDR multiple testing correction (Q < 0.05; Table 2; Additional file 1). Although LD score regression is designed to analyze large sample sizes [18], using this method we nevertheless replicated the directions of five correlations and two of them survived multiple testing correction (Additional file 2). The strongest PRS result was observed for WC_adj_: women with high genetically predicted WC_adj_ have low PTSD risk (PT: 10^–8^; R = –0.079, P < 0.001, Q = 0.011). Additional file 3 shows the distribution of WC_adj_ PRS in PTSD cases and controls. The same variants included in the WC_adj_ PRS also drove four of the other associations observed: waist–hip ratio (WHR, PT = 10^–7^; R = –0.064, *P* = 0.001, Q = 0.023); WHR adjusted for BMI (WHR_adj_, PT = 10^–7^; R = –0.059, *P* = 0.001, Q = 0.030), WHRadj in women aged < 50 years (WHR_adj<50_, PT = 10^–6^; R = –0.076, *P* = 1.25*10^–4^, Q = 0.011), and WHR_adj_ in women aged > 50 years (WHR_adj>50_, PT = 10^–8^; R = –0.065, *P* = 0.001, Q = 0.023). Additional file 4 reports the strong correlations of WC_adj_ PRS with these other anthropometric traits. The association between WC_adj_ PRS and PTSD appears to be specific to women; no correlation was observed in men (Additional file 5).

**Table 2:** Top genetic correlations between PTSD and PRS tested

PRS	PT	SNP N	R	*P* value	Q value
WC_adj_	10^–8^	31	–0.079	6.77 × 10^–5^	0.011
WHR_adj<50_	10^–6^	19	–0.076	1.25 × 10^–4^	0.011
WHR_adj>50_	10^–8^	19	–0.065	0.001	0.023
AFB	0.3	64,579	–0.064	0.001	0.023
WHR	10^–7^	32	–0.064	0.001	0.023
WHR_adj_	10^–7^	51	–0.059	0.003	0.030
Menarche	10^–3^	1510	0.049	0.013	0.074
BMI_<50_	10^–7^	26	0.048	0.015	0.077
HIP	10^–5^	94	0.045	0.024	0.108
Height	0.1	25,369	0.042	0.035	0.144
WC	10^–8^	20	0.034	0.082	0.266
BMI_>50_	10^–5^	104	0.033	0.099	0.306
BMI	10^–4^	316	0.032	0.103	0.312
NEB	10^–6^	2	–0.029	0.141	0.386
HIP_adj_	0.05	13,338	0.029	0.147	0.390
EDU	10^–8^	17	–0.021	0.294	0.641
Menopause	10^–5^	163	0.018	0.360	0.686

Among the significant PRS, the finding related to Age at First Birth (AFB) PRS is the only one that appears to be independent from the WC_adj_ signal because its top correlation is due to the cumulative effects of a large number of variants (AFB top-PT = 0.3, SNP N = 64,579) and not to the effect of a small number of strongly associated loci (WC_adj_ top-PT = 10^–8^, N = 31). We observed that women with a high genetically predicted AFB have low PTSD risk (R = –0.064, *P* = 0.001, Q = 0.023). Additional file 6 shows the distribution of AFB PRS in PTSD cases and controls. No association between AFB PRS and PTSD was observed in men (Additional file 7). Since this genetic overlap appear to be related to the additive effect of a large number of variants (N = 64,579), we investigated which molecular mechanisms are shared between PTSD and AFB. The GO enrichment analysis based on AFB results highlighted several potential molecular mechanisms involved (Q < 0.05; Additional file 8, respectively). Because we observed a large number of significant GO enrichments (Q < 0.05) related to the AFB–PTSD result (N = 40), we conducted a similarity-based network analysis among the significant GO enrichments. This analysis highlighted a GO cluster (Additional file 9) related to multiple signaling mechanisms (e.g. calcium-mediated signaling and MAPK cascade).

**Fig. 1:**
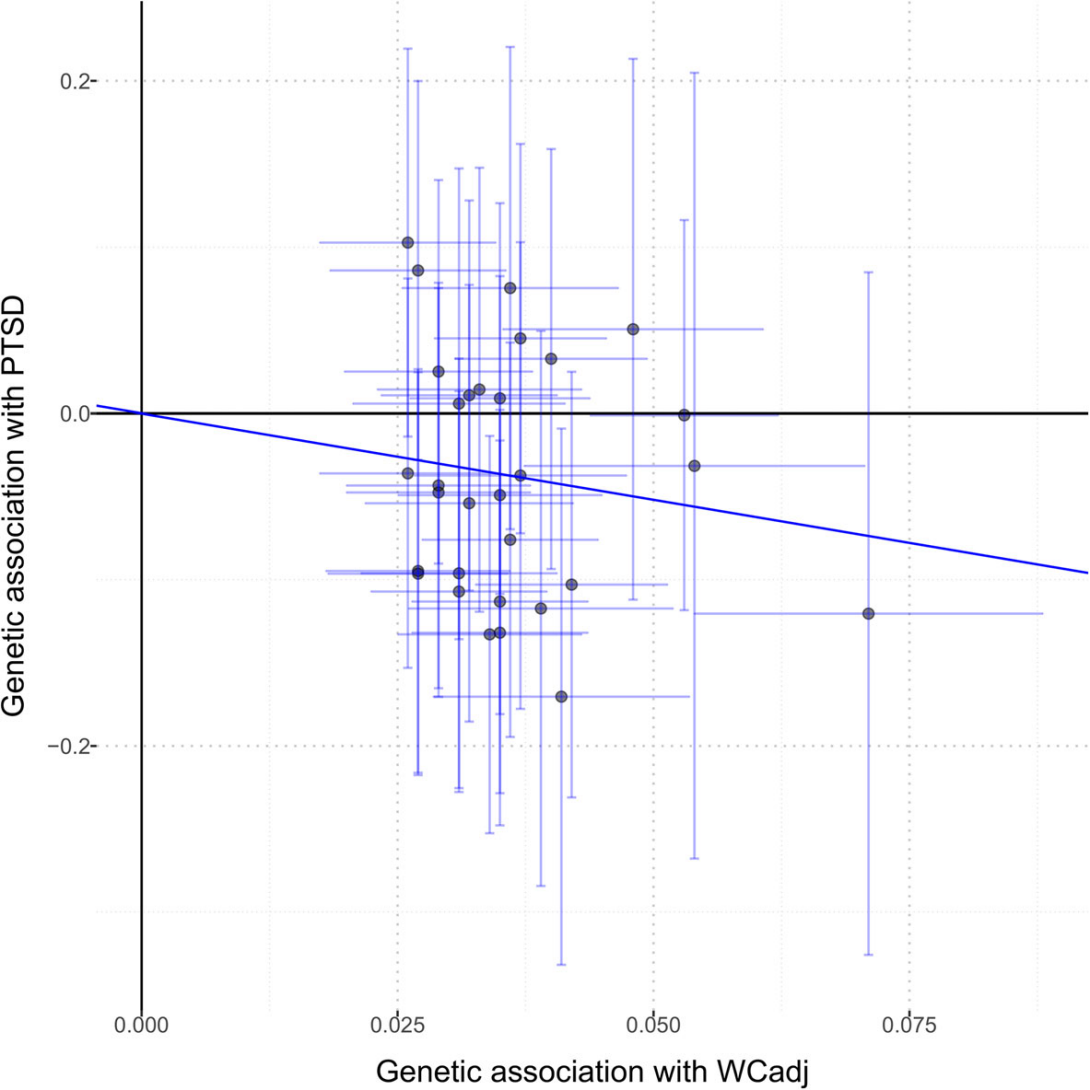
SNP-exposure (WCadj associations) and SNP-outcome (PTSD associations) coefficients used in the MR analysis. *Error bars* (95% CIs) are reported for each association. The *solid line* represents the inverse-variance-weighted estimate

**Fig. 2:**
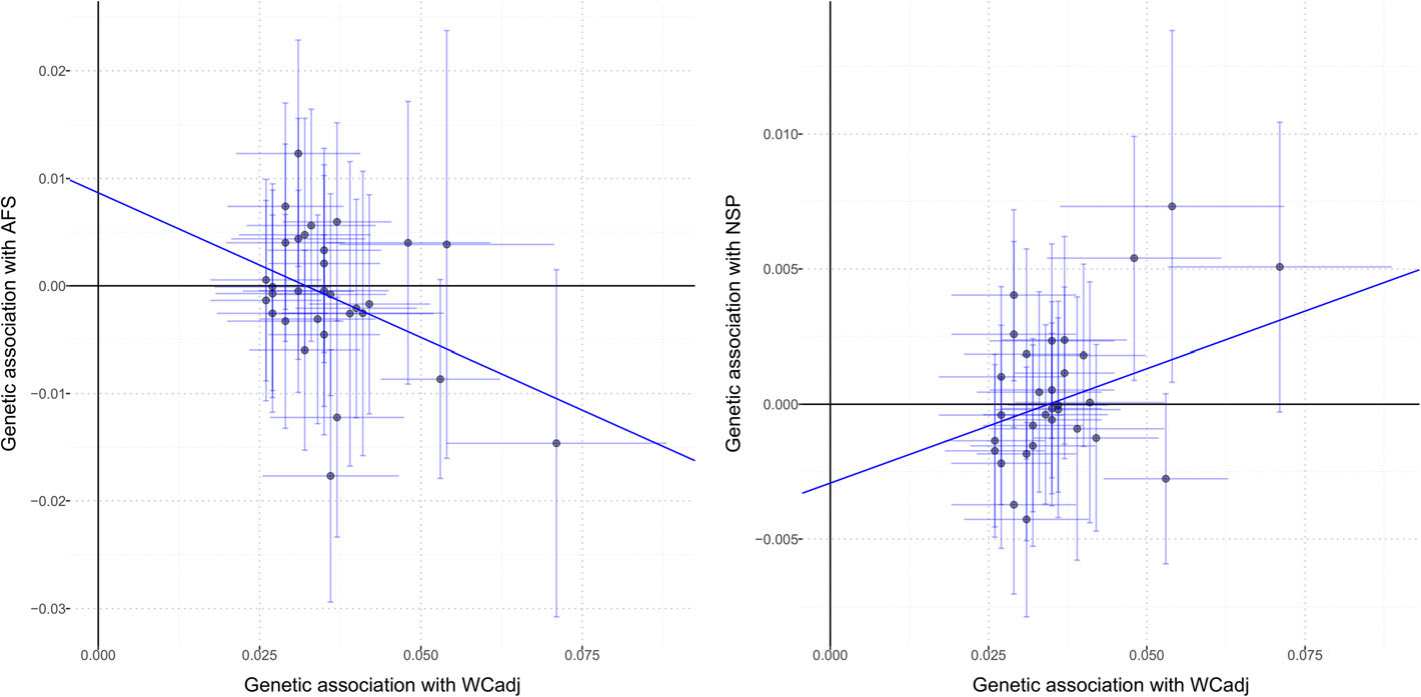
SNP-exposure (WCadj associations) and SNP-outcome (AFS associations, *left*; NSP associations, *right*) coefficients used in the MR analysis. *Error bars* (95% CIs) are reported for each association. The *solid line* represents the MR-Egger estimate

To investigate further the WC_adj_-PTSD genetic correlation, we applied a MR approach to assess the causative mechanisms of this association. We used an inverse-variance-weighted method to calculate an overall estimate of the causal effect considering the coefficients related to the association of these variants with PTSD and WC_adj_ (Fig. 1; Additional file 10). This analysis showed a significant association of effect sizes of WC_adj_-associated SNPs with effect sizes estimated in our PTSD sample (OR = 0.35; 95% confidence interval [CI] = 0.17–0.73; *P* = 0.008). This translated to a relative decrease of 64.6% (95% CI = 27.5–82.7) in the risk of PTSD per 1-SD increase in WC_adj_. This result was consistent considering multiple MR approaches (Additional file 11). Furthermore, the consistency between PRS and MR analyses confirms that there is no sample overlap that is biasing our results. Indeed, these two approaches are susceptible to biases from sample overlaps, but the bias would be in opposite directions: risk of false-positive results in PRS and risk of false-negative results in two-sample MR [43]. Considering the MR-Egger regression intercept, we also observe that the markers did not show evidence of pleiotropic effects (P_pleiotropy_ = 0.979), suggesting that the association of these variants with PTSD is mediated by their effect on body shape. To investigate further the potential causal relationship between PTSD and female body shape, we tested whether the PTSD PRS correlated with WC_adj_. A causal relationship between two traits should show asymmetry in the effect sizes of associated variants [41]. We observed asymmetry between PTSD and WC_adj_: PTSD-associated variants showed no effect on WCadj (*P* > 0.10; Additional file 12) but strongly predicted BMI (*P* = 5.68*10^–7^) and other BMI-related anthropometric traits (Additional file 13). These results support a causal relationship between WC_adj_ and PTSD. We tested whether genetically determined female body shape correlated with sexual behaviors in women using female-specific genetic information regarding AFS and NSP (Additional file 10). Considering a penalized robust MR-Egger regression, we observed associations of WC_adj_ with AFS (Beta = –0.25, SE = 0.10, *P* = 0.013; Fig. 2) and NSP (Beta = 0.16, SE = 0.04, *P* < 0.001; Fig. 2) with significant evidence of pleiotropy in both results (P_pleiotropy_ = 0.017 and < 0.001, respectively), suggesting that the effects of these variants on sexual behaviors could be due to multiple mechanisms.

## Discussion

We found associations of the genetic risk for PTSD in women with genetically determined female body shape and reproductive behaviors. These findings provide novel insight into the mechanisms that may underlie the genetic vulnerability to PTSD in women.

Our strongest result reflects an inverse relationship between genetically determined female body shape and the genetic risk for PTSD. We observed that SNPs associated with anthropometric traits related to body shape independently from BMI (i.e. WC_adj_, WHR, WHR_adj_, WHR_adj<50_, WHR_adj>50_) are linked to the risk for PTSD. No analogous association was present in our male cohort. Human body shape is the result of strong evolutionary pressures, including sexual selection, with evident differences in selection between women and men [44]. The dissimilarities between sexes are largely due to adaptation mechanisms related to female reproductive function [44]. To facilitate childbirth, the pelvis is proportionately larger in women than in men. There is also variability in fat accumulation and distribution that are related to women’s need for energy to be used during pregnancy and nursing: women have a higher percentage of body fat; men accumulate fat in the abdominal region, while women accumulate fat in hips, buttocks, thighs, and the lower abdomen [45]. Consistent with the presence of different regulatory mechanisms related to human body shape, GWAS from the GIANT consortium identified several loci with sex-specific effects and most of them have larger effects in women than in men [30, 32]. Although these sex differences and their regulatory mechanisms are well-known, to our knowledge no large study has investigated the role of genetically determined body shape in women’s mental health. In our study, we observed that a genetically determined evolution-related body shape (i.e. low WC_adj_, WHR, and WHR_adj_) was associated with increased risk of PTSD. A small study reported an association between observational (i.e. phenotypically observed) WHR and PTSD concordant with our finding [46]. Our data are most consistent with a causal relationship between WC_adj_ and PTSD in women. Considering MR-Egger regression intercept, we did not observe a pleiotropic effect between WC_adj_ and PTSD. Conversely, there was an asymmetry in the effect sizes of associated variants: WC_adj_-associated variants affect the risk of PTSD, but PTSD-associated variants do not affect female body shape. Previous evolutionary studies reported that body shape in women is one of the traits related to reproductive attractiveness that has been selected during human evolution [47]. We note that female reproductive attractiveness, which is determined by evolutionary selection pressures, is distinct from socio-cultural norms about female attractiveness that are known to differ over time and place [48]. Our data can be interpreted as supporting that specific anthropometric characteristics may be related to PTSD via their role in increasing the risk of traumatic experiences related to undesired sexual behavior. Further evidence of an apparent causal relationship between WC_adj_ and experiences related to sexual function is provided by the analysis of AFS and NSP. Genetically determined WC_adj_ is associated with AFS and NSP; this provides additional evidence of the relationship between the female body shape and behaviors related to sexual interactions. As indicated by the significant evidence of pleiotropy, the relationship between female body shape and sexual behavior is likely mediated by multiple factors, including behavioral mechanisms and physiological processes related to reproductive function. Unfortunately, we cannot investigate further the relationship between female body shape and traumatic specific experiences, such as sexual assault, because trauma type information is not currently available in the PGC PTSD dataset. We hope to be able to do it in the future. In particular, we believe that longitudinal studies that include detailed life course assessments of traumatic experience will be needed to better understand the role of female body shape in risk of sexual assault or other traumatic event exposure.

Our investigation also identified an association between AFB and PTSD: genetically predicted young maternal AFB is associated with an increase of PTSD risk. This is consistent with epidemiologic observations of an inverse association between maternal AFB and psychiatric distress: mothers giving birth in their teens have an increased risk of a lifetime behavior disorder, PTSD, or an anxiety disorder compared to women who were older at first birth [49]. The AFB–PTSD correlation is due to a genome-wide genetic overlap that includes thousands of independent variants, which is different from the body shape result, which was influenced by a small number of variants. This could indicate that these two traits share some pathways involved in their predisposition. Our enrichment analysis identified several molecular processes. Among them, we observed a large GO cluster of signaling mechanisms that includes several that are known to be involved in the response to traumatic experience, such as calcium-mediated signaling and the MAPK cascade [50, 51]. Observational studies have reported that early trauma exposures are associated with an increased risk of teen pregnancy. In particular, childhood sexual abuse is associated with increased risk for subsequent adolescent pregnancy [52, 53]. In line with this scenario, we note that the AFB GWAS [35] was conducted without excluding birth events from non-consensual sex. For instance, although it accounts for a small fraction of the sample investigated, some of the cohorts included in this previous analysis also included births during childhood. Accordingly, a possible explanation of the AFB–PTSD correlation is that childhood abuse and sexual victimization can contribute to AFB and PTSD, and some of the trauma-response mechanisms involved may be shared among these traits. These mechanisms appear to be different from those related to the WC_adj_ results. The relationship between AFB and PTSD seems to be due to a large genetic overlap (i.e. pleiotropy); WC_adj_ appears to be involved in a causal mechanism related to women’s vulnerability to PTSD (i.e. no pleiotropy).

## Conclusions

In summary, using a genetic approach, we found two associations with the risk of PTSD in women related to body shape and reproductive behaviors. Both mechanisms appear to be female-specific (i.e. there was no such association in men) and they suggest that sex differences in trauma type prevalence and molecular mechanisms of trauma response contribute to the greater vulnerability to PTSD observed in women. Our study demonstrates how genetic research can successfully contribute to the dissection of biological and non-biological mechanisms relevant to women’s health.

## Additional files

Additional file 1: Genetic correlations between PTSD and PRS tested in women. (DOCX 19 kb)

Additional file 2: PTSD genetic correlations tested with LD score regression analysis. (DOCX 12 kb)

Additional file 3: Distribution of WC_adj_ PRS in PTSD cases and controls. (DOCX 333 kb)

Additional file 4: Genetic correlation of WCadj PRS (SNP N = 31, PT =10^–8^) with the other anthropometric traits associated with PTSD in women. (DOCX 12 kb)

Additional file 5: Correlation between WC_adj_ PRS and PTSD in men. (DOCX 12 kb)

Additional file 6: Distribution of AFB PRS in PTSD cases and controls. (DOCX 361 kb)

Additional file 7: Correlation between AFB PRS and PTSD in men. (DOCX 12 kb)

Additional file 8: Gene Ontology (GO) enrichments (Q value < 0.05) for variants with AFB GWAS *P* < 0.3 and effect direction concordant with the AFB-PTSD correlation. (DOCX 14 kb)

Additional file 9: Similarity network based on GO enrichment results related to the AFB-PTSD correlation. Bubble color indicates the GO *P* value; bubble size indicates the frequency of the GO term in the Gene Ontology Annotation (UniProt-GOA) Database which annotates all UniProt entries with GO terms. Highly similar GO terms are linked by edges in the graph, where the line width indicates the degree of similarity. (DOCX 179 kb)

Additional file 10: SNP-exposure and SNP-outcome coefficients of the variants in included in the WC_adj_ PRS. (DOCX 15 kb)

Additional file 11: SNP-exposure (WCadj associations) and SNP-outcome (PTSD associations) coefficients used in the MR analysis. The lines (see legend) represents the results observed considering different methods. (DOCX 383 kb)

Additional file 12: Correlation between PTSD PRS and WC_adj_. (DOCX 12 kb)

Additional file 13: Top results of the reverse PRS analysis of PTSD with respect to the main anthropometric traits. (DOCX 12 kb)

## Abbreviations

AFB: Age at first birth
AFS: Age at first sexual intercourse
BMI: Body mass index
FDR: False discovery rate
GO: Gene Ontology
GWAS: Genome-wide association study
MR: Mendelian randomization
NSP: Number of sexual partners
PRS: Polygenic risk score
PTSD: Posttraumatic stress disorder
SNP: single nucleotide polymorphism
WC_adj_: BMI-adjusted waist circumference
WHR: Waist–hip ratio
WHR_adj_: WHR adjusted for BMI
WHR_adj<50_: WHRadj in women aged < 50 years
WHR_adj>50_: WHR_adj_ in women aged > 50 years
