# Supplementary material for: A putative causal relationship between genetically determined female body shape and posttraumatic stress disorder
Source: Genome Med. 2017 Nov 27;9:99. doi: 10.1186/s13073-017-0491-4 (PMC5702961; doi:10.1186/s13073-017-0491-4)
Supplement: Supplementary file 1 — Genetic correlations between PTSD and PRS tested in women. (DOCX 19 kb) [file 13073_2017_491_MOESM1_ESM.docx]

**Additional File 1**: Genetic correlations between PTSD and PRS tested in women.

| **GWAS** | **PT** | **SNP N** | **R^2^** | **P value** | **Q value** |
| --- | --- | --- | --- | --- | --- |
| WCadj | 1.00E-08 | 31 | 0.006 | 6.77E-05 | 0.011 |
| WHRadjLE50 | 1.00E-06 | 19 | 0.006 | 1.25E-04 | 0.011 |
| WCadj | 1.00E-07 | 39 | 0.005 | 6.62E-04 | 0.023 |
| WHRadjLE50 | 5.00E-08 | 13 | 0.004 | 7.82E-04 | 0.023 |
| WHRadjLE50 | 1.00E-07 | 13 | 0.004 | 7.82E-04 | 0.023 |
| WHRadjGT50 | 1.00E-08 | 19 | 0.004 | 1.08E-03 | 0.023 |
| AFB | 3.00E-01 | 64579 | 0.004 | 1.15E-03 | 0.023 |
| WHR | 1.00E-07 | 32 | 0.004 | 1.28E-03 | 0.023 |
| WHR | 1.00E-06 | 46 | 0.004 | 1.30E-03 | 0.023 |
| WHRadjLE50 | 1.00E-08 | 12 | 0.004 | 1.33E-03 | 0.023 |
| WHRadjGT50 | 1.00E-05 | 70 | 0.004 | 1.80E-03 | 0.025 |
| WHRadjGT50 | 1.00E-06 | 39 | 0.004 | 1.83E-03 | 0.025 |
| WHRadjGT50 | 5.00E-08 | 26 | 0.004 | 2.03E-03 | 0.025 |
| WHR | 1.00E-08 | 25 | 0.004 | 2.17E-03 | 0.025 |
| WCadj | 1.00E-06 | 63 | 0.004 | 2.29E-03 | 0.025 |
| AFB | 5.00E-01 | 94351 | 0.004 | 2.37E-03 | 0.025 |
| WHRadj | 1.00E-07 | 51 | 0.003 | 3.10E-03 | 0.030 |
| WHRadj | 1.00E-06 | 67 | 0.003 | 3.34E-03 | 0.030 |
| WHRadj | 1.00E-04 | 221 | 0.003 | 3.56E-03 | 0.030 |
| AFB | 5.00E-02 | 15568 | 0.003 | 3.67E-03 | 0.030 |
| WHRadjGT50 | 1.00E-07 | 27 | 0.003 | 3.78E-03 | 0.030 |
| WHR | 1.00E-04 | 177 | 0.003 | 3.91E-03 | 0.030 |
| WHRadjLE50 | 1.00E-05 | 32 | 0.003 | 4.25E-03 | 0.031 |
| WHRadjGT50 | 1.00E-04 | 139 | 0.003 | 6.91E-03 | 0.049 |
| WHR | 1.00E-05 | 72 | 0.003 | 7.86E-03 | 0.053 |
| AFB | 1.00E-01 | 27008 | 0.003 | 8.97E-03 | 0.057 |
| WHRadj | 1.00E-08 | 38 | 0.003 | 9.01E-03 | 0.057 |
| AFB | 1.00E-07 | 6 | 0.003 | 1.04E-02 | 0.063 |
| WHRadjLE50 | 1.00E-01 | 25323 | 0.003 | 1.09E-02 | 0.064 |
| WHRadj | 1.00E-05 | 107 | 0.002 | 1.27E-02 | 0.072 |
| Menarche | 1.00E-03 | 1510 | 0.002 | 1.34E-02 | 0.074 |
| BMIle50 | 1.00E-07 | 26 | 0.002 | 1.45E-02 | 0.077 |
| WHRadjLE50 | 5.00E-02 | 13975 | 0.002 | 1.60E-02 | 0.080 |
| BMIle50 | 1.00E-08 | 17 | 0.002 | 1.60E-02 | 0.080 |
| WHRadj | 1.00E-03 | 730 | 0.002 | 1.80E-02 | 0.087 |
| AFB | 1.00E-06 | 11 | 0.002 | 2.26E-02 | 0.107 |
| HIP | 1.00E-05 | 94 | 0.002 | 2.35E-02 | 0.108 |
| HIP | 1.00E-04 | 215 | 0.002 | 2.80E-02 | 0.125 |
| HIP | 1.00E-06 | 49 | 0.002 | 3.24E-02 | 0.141 |
| WCadj | 1.00E-05 | 115 | 0.002 | 3.41E-02 | 0.144 |
| Height | 1.00E-01 | 25369 | 0.002 | 3.48E-02 | 0.144 |
| BMIle50 | 1.00E-05 | 87 | 0.002 | 3.73E-02 | 0.151 |
| Height | 3.00E-01 | 63333 | 0.002 | 4.93E-02 | 0.191 |
| WHRadjGT50 | 1.00E-03 | 533 | 0.002 | 4.97E-02 | 0.191 |
| WHRadjLE50 | 1.00E-04 | 79 | 0.001 | 5.05E-02 | 0.191 |
| WCadj | 1.00E-03 | 703 | 0.001 | 5.59E-02 | 0.207 |
| HIP | 1.00E-07 | 31 | 0.001 | 5.81E-02 | 0.210 |
| WCadj | 1.00E-04 | 252 | 0.001 | 6.74E-02 | 0.239 |
| BMIle50 | 1.00E-06 | 45 | 0.001 | 7.11E-02 | 0.247 |
| WHR | 1.00E-03 | 646 | 0.001 | 7.36E-02 | 0.250 |
| HIP | 1.00E-03 | 708 | 0.001 | 8.14E-02 | 0.266 |
| WC | 1.00E-08 | 20 | 0.001 | 8.15E-02 | 0.266 |
| AFB | 1.00E-08 | 3 | 0.001 | 8.95E-02 | 0.287 |
| WHRadjLE50 | 3.00E-01 | 61860 | 0.001 | 9.76E-02 | 0.306 |
| BMIgt50 | 1.00E-05 | 104 | 0.001 | 9.91E-02 | 0.306 |
| BMI | 1.00E-04 | 316 | 0.001 | 1.03E-01 | 0.312 |
| Height | 5.00E-01 | 94244 | 0.001 | 1.07E-01 | 0.320 |
| Height | 5.00E-02 | 14130 | 0.001 | 1.10E-01 | 0.323 |
| HIP | 1.00E-08 | 22 | 0.001 | 1.23E-01 | 0.354 |
| WHRadjLE50 | 5.00E-01 | 91408 | 0.001 | 1.28E-01 | 0.363 |
| Menarche | 1.00E-07 | 101 | 0.001 | 1.32E-01 | 0.367 |
| NCB | 1.00E-06 | 2 | 0.001 | 1.41E-01 | 0.386 |
| NCB | 1.00E-04 | 66 | 0.001 | 1.45E-01 | 0.390 |
| HIPadj | 5.00E-02 | 13338 | 0.001 | 1.47E-01 | 0.390 |
| BMI | 1.00E-08 | 44 | 0.001 | 1.72E-01 | 0.450 |
| WC | 1.00E-07 | 29 | 0.001 | 1.77E-01 | 0.455 |
| NCB | 5.00E-02 | 13635 | 0.001 | 1.79E-01 | 0.455 |
| AFB | 1.00E-04 | 131 | 0.001 | 2.43E-01 | 0.604 |
| Menarche | 1.00E-06 | 152 | 0.001 | 2.52E-01 | 0.604 |
| BMIgt50 | 1.00E-08 | 17 | 0.001 | 2.53E-01 | 0.604 |
| BMI | 1.00E-07 | 57 | 0.001 | 2.54E-01 | 0.604 |
| BMI | 5.00E-02 | 12920 | <0.001 | 2.61E-01 | 0.604 |
| WC | 1.00E-06 | 41 | <0.001 | 2.64E-01 | 0.604 |
| Menarche | 5.00E-02 | 18141 | <0.001 | 2.65E-01 | 0.604 |
| HIPadj | 1.00E-01 | 24128 | <0.001 | 2.66E-01 | 0.604 |
| AFB | 1.00E-03 | 678 | <0.001 | 2.71E-01 | 0.606 |
| WC | 1.00E-05 | 76 | <0.001 | 2.82E-01 | 0.622 |
| EDU | 1.00E-08 | 17 | <0.001 | 2.94E-01 | 0.641 |
| EDU | 1.00E-03 | 1678 | <0.001 | 3.06E-01 | 0.658 |
| BMIgt50 | 1.00E-07 | 24 | <0.001 | 3.24E-01 | 0.686 |
| BMI | 3.00E-01 | 59079 | <0.001 | 3.29E-01 | 0.686 |
| WHRadjGT50 | 5.00E-02 | 13129 | <0.001 | 3.41E-01 | 0.686 |
| BMI | 1.00E-05 | 142 | <0.001 | 3.52E-01 | 0.686 |
| EDU | 1.00E-05 | 144 | <0.001 | 3.59E-01 | 0.686 |
| Menopause | 1.00E-05 | 163 | <0.001 | 3.60E-01 | 0.686 |
| HIPadj | 1.00E-07 | 57 | <0.001 | 3.60E-01 | 0.686 |
| BMIle50 | 1.00E-03 | 854 | <0.001 | 3.62E-01 | 0.686 |
| WC | 5.00E-01 | 89940 | <0.001 | 3.66E-01 | 0.686 |
| EDU | 1.00E-04 | 425 | <0.001 | 3.73E-01 | 0.686 |
| Menopause | 1.00E-04 | 305 | <0.001 | 3.75E-01 | 0.686 |
| BMIle50 | 1.00E-04 | 219 | <0.001 | 3.75E-01 | 0.686 |
| BMI | 1.00E-01 | 23141 | <0.001 | 3.78E-01 | 0.686 |
| BMI | 1.00E-03 | 897 | <0.001 | 3.80E-01 | 0.686 |
| BMIgt50 | 1.00E-06 | 47 | <0.001 | 3.83E-01 | 0.686 |
| Menarche | 1.00E-08 | 76 | <0.001 | 3.83E-01 | 0.686 |
| NCB | 1.00E-05 | 6 | <0.001 | 3.91E-01 | 0.692 |
| BMI | 5.00E-01 | 89780 | <0.001 | 4.14E-01 | 0.722 |
| WHR | 5.00E-02 | 13312 | <0.001 | 4.18E-01 | 0.722 |
| Height | 1.00E-03 | 1071 | <0.001 | 4.20E-01 | 0.722 |
| BMI | 1.00E-06 | 91 | <0.001 | 4.27E-01 | 0.725 |
| WCadj | 5.00E-01 | 91646 | <0.001 | 4.41E-01 | 0.743 |
| HIPadj | 1.00E-08 | 44 | <0.001 | 4.48E-01 | 0.747 |
| WHR | 1.00E-01 | 24190 | <0.001 | 4.60E-01 | 0.759 |
| EDU | 1.00E-07 | 28 | <0.001 | 4.67E-01 | 0.763 |
| HIPadj | 1.00E-06 | 83 | <0.001 | 4.75E-01 | 0.767 |
| HIPadj | 1.00E-03 | 836 | <0.001 | 4.78E-01 | 0.767 |
| WCadj | 3.00E-01 | 60896 | <0.001 | 5.05E-01 | 0.796 |
| WC | 3.00E-01 | 58962 | <0.001 | 5.07E-01 | 0.796 |
| BMIle50 | 5.00E-01 | 92694 | <0.001 | 5.13E-01 | 0.796 |
| WHR | 5.00E-01 | 90931 | <0.001 | 5.34E-01 | 0.796 |
| Menarche | 1.00E-04 | 544 | <0.001 | 5.38E-01 | 0.796 |
| BMIgt50 | 1.00E-03 | 939 | <0.001 | 5.43E-01 | 0.796 |
| Menopause | 3.00E-01 | 61914 | <0.001 | 5.48E-01 | 0.796 |
| Menopause | 1.00E-01 | 25888 | <0.001 | 5.51E-01 | 0.796 |
| BMIle50 | 3.00E-01 | 63593 | <0.001 | 5.51E-01 | 0.796 |
| Height | 1.00E-07 | 89 | <0.001 | 5.55E-01 | 0.796 |
| BMIle50 | 1.00E-01 | 27005 | <0.001 | 5.58E-01 | 0.796 |
| WCadj | 5.00E-02 | 13565 | <0.001 | 5.60E-01 | 0.796 |
| Height | 1.00E-06 | 145 | <0.001 | 5.62E-01 | 0.796 |
| Menarche | 1.00E-05 | 267 | <0.001 | 5.65E-01 | 0.796 |
| HIPadj | 1.00E-05 | 148 | <0.001 | 5.66E-01 | 0.796 |
| WC | 1.00E-04 | 188 | <0.001 | 5.78E-01 | 0.800 |
| Menopause | 5.00E-01 | 91166 | <0.001 | 5.79E-01 | 0.800 |
| BMIgt50 | 1.00E-04 | 256 | <0.001 | 5.88E-01 | 0.806 |
| Menarche | 1.00E-01 | 29980 | <0.001 | 6.45E-01 | 0.877 |
| EDU | 1.00E-01 | 42862 | <0.001 | 6.63E-01 | 0.892 |
| HIPadj | 5.00E-01 | 90722 | <0.001 | 6.66E-01 | 0.892 |
| Menopause | 5.00E-02 | 14597 | <0.001 | 6.76E-01 | 0.898 |
| NCB | 5.00E-01 | 92050 | <0.001 | 6.89E-01 | 0.906 |
| Height | 1.00E-04 | 451 | <0.001 | 6.94E-01 | 0.906 |
| HIP | 5.00E-02 | 13040 | <0.001 | 7.03E-01 | 0.906 |
| WHRadjGT50 | 1.00E-01 | 23947 | <0.001 | 7.03E-01 | 0.906 |
| WC | 5.00E-02 | 12796 | <0.001 | 7.32E-01 | 0.919 |
| EDU | 5.00E-02 | 25665 | <0.001 | 7.42E-01 | 0.919 |
| WHRadj | 5.00E-01 | 91228 | <0.001 | 7.45E-01 | 0.919 |
| HIPadj | 1.00E-04 | 301 | <0.001 | 7.46E-01 | 0.919 |
| BMIgt50 | 1.00E-01 | 27410 | <0.001 | 7.50E-01 | 0.919 |
| EDU | 1.00E-06 | 60 | <0.001 | 7.53E-01 | 0.919 |
| HIP | 1.00E-01 | 23729 | <0.001 | 7.77E-01 | 0.919 |
| WC | 1.00E-01 | 23213 | <0.001 | 7.82E-01 | 0.919 |
| WHRadj | 1.00E-01 | 24214 | <0.001 | 7.83E-01 | 0.919 |
| HIP | 3.00E-01 | 59660 | <0.001 | 7.86E-01 | 0.919 |
| BMIgt50 | 5.00E-01 | 91954 | <0.001 | 7.90E-01 | 0.919 |
| Menopause | 1.00E-03 | 880 | <0.001 | 7.91E-01 | 0.919 |
| EDU | 5.00E-01 | 140594 | <0.001 | 7.94E-01 | 0.919 |
| EDU | 3.00E-01 | 97789 | <0.001 | 7.94E-01 | 0.919 |
| AFB | 1.00E-05 | 35 | <0.001 | 7.95E-01 | 0.919 |
| WC | 1.00E-03 | 601 | <0.001 | 8.10E-01 | 0.920 |
| WHR | 3.00E-01 | 60468 | <0.001 | 8.16E-01 | 0.920 |
| NCB | 1.00E-01 | 24815 | <0.001 | 8.17E-01 | 0.920 |
| BMIgt50 | 5.00E-02 | 16029 | <0.001 | 8.28E-01 | 0.920 |
| NCB | 1.00E-03 | 489 | <0.001 | 8.28E-01 | 0.920 |
| NCB | 3.00E-01 | 61977 | <0.001 | 8.28E-01 | 0.920 |
| Menarche | 3.00E-01 | 65872 | <0.001 | 8.65E-01 | 0.949 |
| HIPadj | 3.00E-01 | 60171 | <0.001 | 8.65E-01 | 0.949 |
| WHRadjGT50 | 3.00E-01 | 59728 | <0.001 | 8.72E-01 | 0.950 |
| WHRadj | 3.00E-01 | 60734 | <0.001 | 8.97E-01 | 0.970 |
| WCadj | 1.00E-01 | 24524 | <0.001 | 9.05E-01 | 0.970 |
| Height | 1.00E-08 | 68 | <0.001 | 9.09E-01 | 0.970 |
| WHRadjGT50 | 5.00E-01 | 88842 | <0.001 | 9.16E-01 | 0.970 |
| HIP | 5.00E-01 | 90081 | <0.001 | 9.18E-01 | 0.970 |
| WHRadjLE50 | 1.00E-03 | 519 | <0.001 | 9.28E-01 | 0.974 |
| BMIgt50 | 3.00E-01 | 63042 | <0.001 | 9.49E-01 | 0.983 |
| Menarche | 5.00E-01 | 94351 | <0.001 | 9.50E-01 | 0.983 |
| Menopause | 1.00E-07 | 72 | <0.001 | 9.58E-01 | 0.983 |
| Menopause | 1.00E-08 | 55 | <0.001 | 9.60E-01 | 0.983 |
| Menopause | 1.00E-06 | 101 | <0.001 | 9.67E-01 | 0.984 |
| Height | 1.00E-05 | 254 | <0.001 | 9.78E-01 | 0.989 |
| BMIle50 | 5.00E-02 | 15646 | <0.001 | 9.86E-01 | 0.990 |
| WHRadj | 5.00E-02 | 13413 | <0.001 | 9.90E-01 | 0.990 |
