# Supplementary material for: A putative causal relationship between genetically determined female body shape and posttraumatic stress disorder
Source: Genome Med. 2017 Nov 27;9:99. doi: 10.1186/s13073-017-0491-4 (PMC5702961; doi:10.1186/s13073-017-0491-4)
Supplement: Supplementary file 2 — PTSD genetic correlations tested with LD score regression analysis. (DOCX 12 kb) [file 13073_2017_491_MOESM2_ESM.docx]

**Additional File 2:** PTSD genetic correlations tested with LD score regression analysis.

| **Trait** | **rg** | **se** | **p** |
| --- | --- | --- | --- |
| AFB | -0.3739 | 0.1011 | 0.0002 |
| WHR_adj>50_ | -0.2517 | 0.0972 | 0.0096 |
| WHR_adj_ | -0.1348 | 0.0876 | 0.124 |
| WC_adj_ | -0.0469 | 0.0817 | 0.5663 |
| WHR | -0.0473 | 0.0829 | 0.568 |
| WHR_adj<50_ | 0.0565 | 0.1402 | 0.687 |
