# Supplementary figures and images for: A putative causal relationship between genetically determined female body shape and posttraumatic stress disorder
Source: Genome Med. 2017 Nov 27;9:99. doi: 10.1186/s13073-017-0491-4 (PMC5702961; doi:10.1186/s13073-017-0491-4)

**
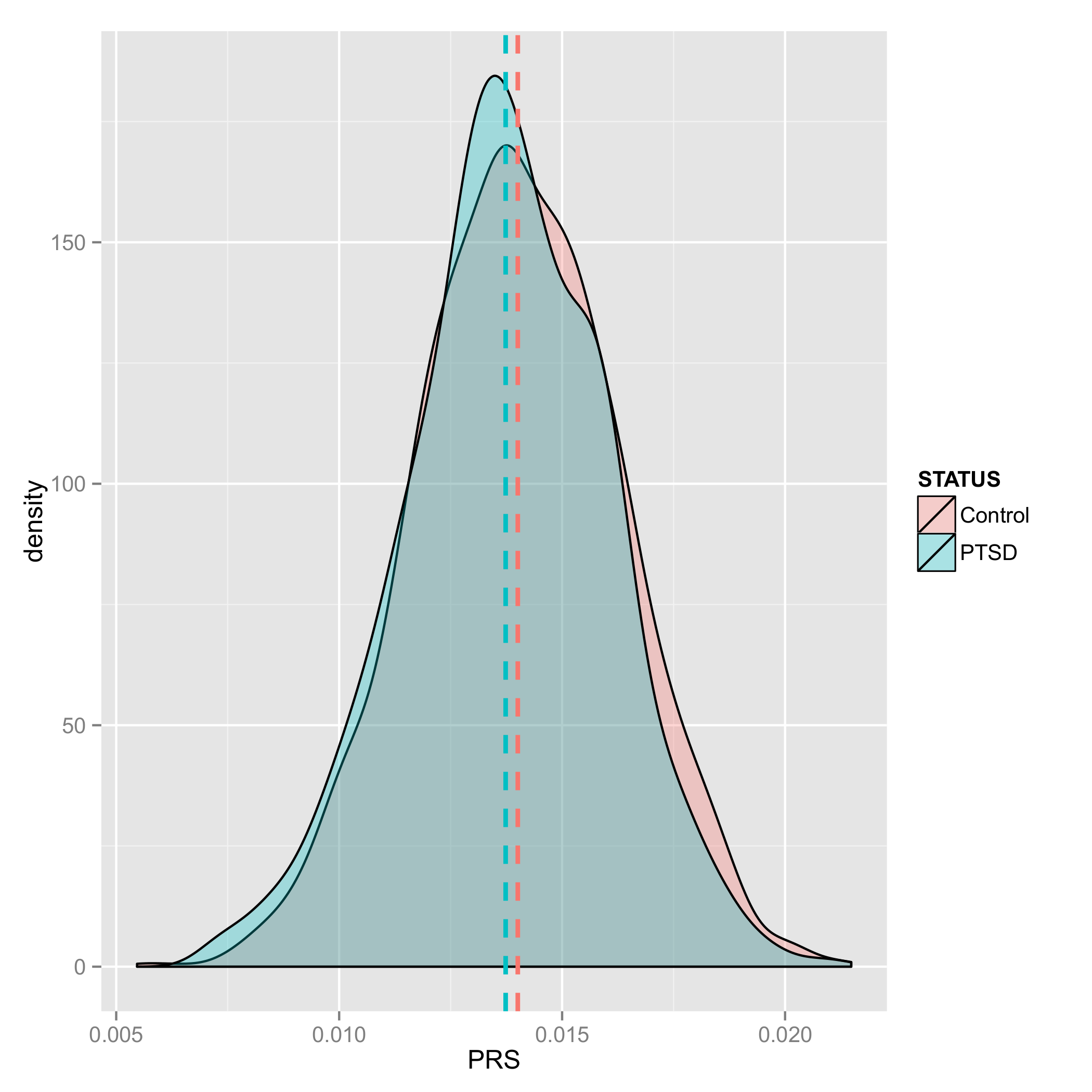
**

**Additional File 3:** Distribution of WCadj PRS in PTSD cases and controls.

Supplement: Supplementary file 3 — Distribution of WCadj PRS in PTSD cases and controls. (DOCX 333 kb) [file 13073_2017_491_MOESM3_ESM.docx]

**
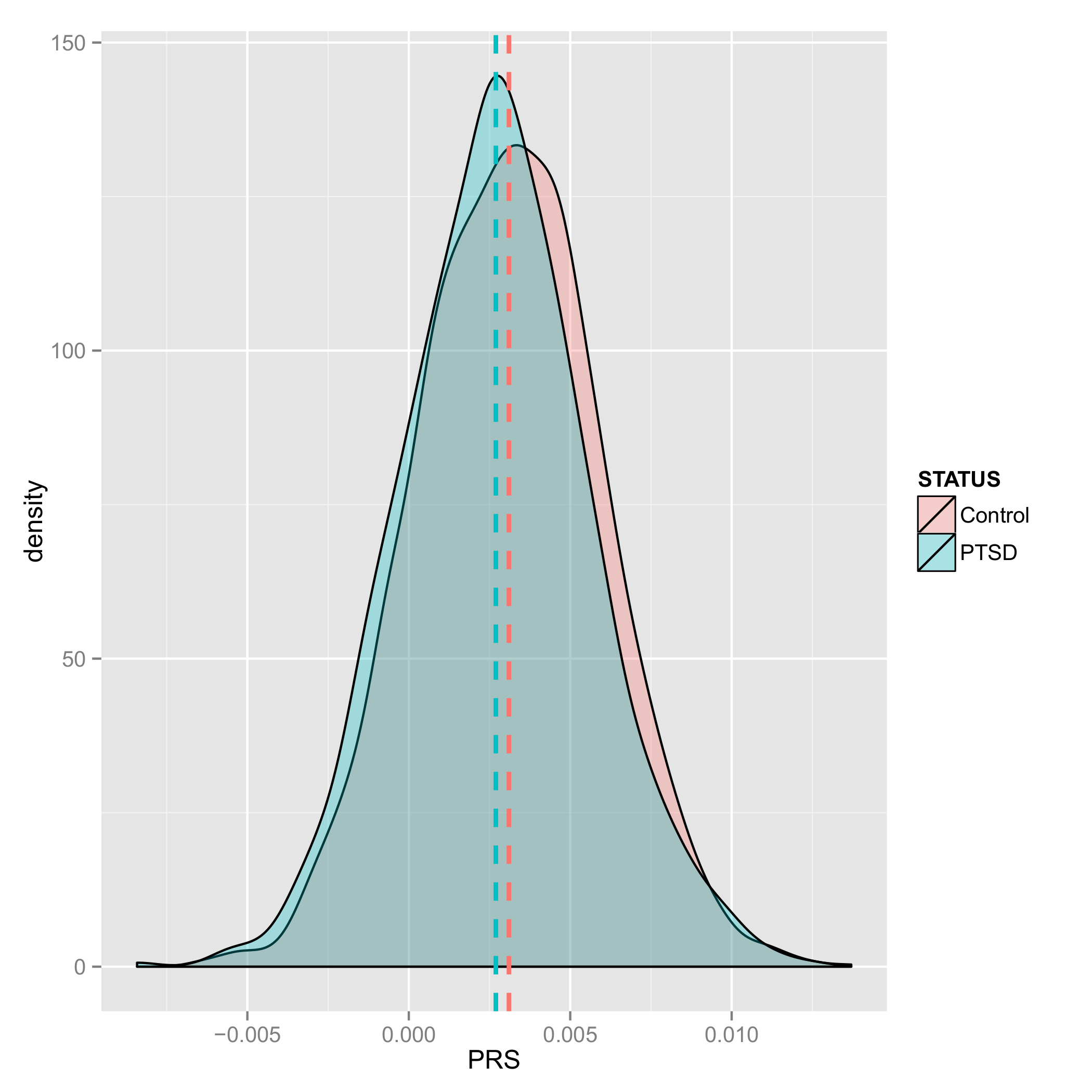
**

**Additional File 6:** Distribution of AFB PRS in PTSD cases and controls.

Supplement: Supplementary file 6 — Distribution of AFB PRS in PTSD cases and controls. (DOCX 361 kb) [file 13073_2017_491_MOESM6_ESM.docx]
