# Supplementary material for: A putative causal relationship between genetically determined female body shape and posttraumatic stress disorder
Source: Genome Med. 2017 Nov 27;9:99. doi: 10.1186/s13073-017-0491-4 (PMC5702961; doi:10.1186/s13073-017-0491-4)
Supplement: Supplementary file 4 — Genetic correlation of WCadj PRS (SNP N = 31, PT =10–8) with the other anthropometric traits associated with PTSD in women. (DOCX 12 kb) [file 13073_2017_491_MOESM4_ESM.docx]

**Additional File 4:** Genetic correlation of WCadj PRS (SNP N = 31, PT =10^-8^) with the other anthropometric traits associated with PTSD in women.

| **GWAS** | **R^2^** | **P value** |
| --- | --- | --- |
| WHRadj | 0.018 | 2.16E-288 |
| WHR | 0.011 | 2.05E-196 |
| WHRadjGT50 | 0.011 | 1.10E-164 |
| WHRadjLE50 | 0.017 | 4.23E-122 |
