# Supplementary material for: A putative causal relationship between genetically determined female body shape and posttraumatic stress disorder
Source: Genome Med. 2017 Nov 27;9:99. doi: 10.1186/s13073-017-0491-4 (PMC5702961; doi:10.1186/s13073-017-0491-4)
Supplement: Supplementary file 5 — Correlation between WCadj PRS and PTSD in men. (DOCX 12 kb) [file 13073_2017_491_MOESM5_ESM.docx]

**Additional File 5:** Correlation between WC_adj_ PRS and PTSD in men.

| **PT** | **nsnps** | **R^2^** | **P value** |
| --- | --- | --- | --- |
| 1.00E-08 | 26 | 0.00015 | 0.555 |
| 1.00E-07 | 48 | 0.00045 | 0.310 |
| 1.00E-06 | 79 | 0.00006 | 0.706 |
| 1.00E-05 | 125 | 0.00016 | 0.544 |
| 1.00E-04 | 259 | 0.00064 | 0.223 |
| 1.00E-03 | 867 | 0.00065 | 0.221 |
| 5.00E-02 | 16942 | 0.00034 | 0.379 |
| 1.00E-01 | 30683 | 0.00007 | 0.691 |
| 3.00E-01 | 76264 | 0.00006 | 0.717 |
| 5.00E-01 | 114761 | 0.00003 | 0.781 |
