# Supplementary material for: A putative causal relationship between genetically determined female body shape and posttraumatic stress disorder
Source: Genome Med. 2017 Nov 27;9:99. doi: 10.1186/s13073-017-0491-4 (PMC5702961; doi:10.1186/s13073-017-0491-4)
Supplement: Supplementary file 7 — Correlation between AFB PRS and PTSD in men. (DOCX 12 kb) [file 13073_2017_491_MOESM7_ESM.docx]

**Additional File 7:** Correlation between AFB PRS and PTSD in men.

| **PT** | **SNP N** | **R2** | **P value** |
| --- | --- | --- | --- |
| 1.00E-06 | 1 | 0.00024 | 0.457 |
| 1.00E-05 | 7 | 0.00017 | 0.527 |
| 1.00E-04 | 70 | 0.00129 | 0.085 |
| 1.00E-03 | 631 | 0.00039 | 0.346 |
| 5.00E-02 | 18982 | 0.00043 | 0.319 |
| 1.00E-01 | 34617 | 0.00016 | 0.542 |
| 3.00E-01 | 87423 | 0.00004 | 0.754 |
| 5.00E-01 | 129892 | 0.00010 | 0.630 |
