# Supplementary material for: A putative causal relationship between genetically determined female body shape and posttraumatic stress disorder
Source: Genome Med. 2017 Nov 27;9:99. doi: 10.1186/s13073-017-0491-4 (PMC5702961; doi:10.1186/s13073-017-0491-4)
Supplement: Supplementary file 8 — Gene Ontology (GO) enrichments (Q value < 0.05) for variants with AFB GWAS P < 0.3 and effect direction concordant with the AFB-PTSD correlation. (DOCX 14 kb) [file 13073_2017_491_MOESM8_ESM.docx]

**Additional File 8:** Gene Ontology (GO) enrichments (Q value < 0.05) for variants with AFB GWAS p < 0.3 and effect direction concordant with the AFB-PTSD correlation.

| **GO term** | **Fold Enrichment** | **P value** | **Q value** |
| --- | --- | --- | --- |
| cellular process (GO:0009987) | 1.08 | 7.01E-12 | 1.33E-09 |
| metabolic process (GO:0008152) | 1.08 | 3.24E-10 | 3.08E-08 |
| intracellular signal transduction (GO:0035556) | 1.25 | 1.07E-09 | 6.78E-08 |
| developmental process (GO:0032502) | 1.15 | 1.53E-07 | 7.27E-06 |
| primary metabolic process (GO:0044238) | 1.07 | 3.11E-07 | 1.18E-05 |
| cellular component morphogenesis (GO:0032989) | 1.23 | 3.18E-05 | 0.001007 |
| phosphate-containing compound metabolic process (GO:0006796) | 1.14 | 4.90E-05 | 0.00133 |
| cell communication (GO:0007154) | 1.09 | 6.99E-05 | 0.001660125 |
| cellular component movement (GO:0006928) | 1.24 | 1.42E-04 | 0.002997778 |
| mesoderm development (GO:0007498) | 1.22 | 2.24E-04 | 0.003886364 |
| signal transduction (GO:0007165) | 1.09 | 2.25E-04 | 0.003886364 |
| catabolic process (GO:0009056) | 1.16 | 2.74E-04 | 0.004338333 |
| nucleobase-containing compound metabolic process (GO:0006139) | 1.07 | 3.08E-04 | 0.004501538 |
| nitrogen compound metabolic process (GO:0006807) | 1.09 | 4.46E-04 | 0.006052857 |
| intracellular protein transport (GO:0006886) | 1.13 | 5.89E-04 | 0.00722 |
| protein transport (GO:0015031) | 1.13 | 6.08E-04 | 0.00722 |
| system development (GO:0048731) | 1.13 | 6.52E-04 | 0.007287059 |
| death (GO:0016265) | 1.21 | 7.74E-04 | 0.00774 |
| cell death (GO:0008219) | 1.21 | 7.74E-04 | 0.00774 |
| apoptotic process (GO:0006915) | 1.21 | 8.91E-04 | 0.0084645 |
| cell cycle (GO:0007049) | 1.13 | 1.08E-03 | 0.009771429 |
| homeostatic process (GO:0042592) | 1.28 | 1.18E-03 | 0.01019091 |
| nervous system development (GO:0007399) | 1.15 | 2.02E-03 | 0.01668696 |
| mitosis (GO:0007067) | 1.19 | 2.76E-03 | 0.02185 |
| visual perception (GO:0007601) | 1.27 | 3.38E-03 | 0.02378519 |
| cell adhesion (GO:0007155) | 1.17 | 3.28E-03 | 0.02378519 |
| biological adhesion (GO:0022610) | 1.17 | 3.28E-03 | 0.02378519 |
| MAPK cascade (GO:0000165) | 1.2 | 3.57E-03 | 0.024225 |
| cellular protein modification process (GO:0006464) | 1.11 | 3.75E-03 | 0.02456897 |
| calcium-mediated signaling (GO:0019722) | 1.4 | 4.43E-03 | 0.02619697 |
| muscle contraction (GO:0006936) | 1.29 | 4.37E-03 | 0.02619697 |
| lipid metabolic process (GO:0006629) | 1.15 | 4.46E-03 | 0.02619697 |
| transport (GO:0006810) | 1.07 | 4.55E-03 | 0.02619697 |
| sensory perception of sound (GO:0007605) | 1.44 | 4.88E-03 | 0.02727059 |
| heart development (GO:0007507) | 1.3 | 5.34E-03 | 0.02828889 |
| localization (GO:0051179) | 1.07 | 5.36E-03 | 0.02828889 |
| transmembrane receptor protein tyrosine kinase signaling pathway (GO:0007169) | 1.24 | 5.96E-03 | 0.03060541 |
| cell-cell signaling (GO:0007267) | 1.15 | 6.82E-03 | 0.0341 |
| RNA metabolic process (GO:0016070) | 1.07 | 7.57E-03 | 0.03687949 |
| protein metabolic process (GO:0019538) | 1.07 | 8.63E-03 | 0.0409925 |
