# Supplementary material for: A putative causal relationship between genetically determined female body shape and posttraumatic stress disorder
Source: Genome Med. 2017 Nov 27;9:99. doi: 10.1186/s13073-017-0491-4 (PMC5702961; doi:10.1186/s13073-017-0491-4)
Supplement: Supplementary file 9 — Similarity network based on GO enrichment results related to the AFB-PTSD correlation. Bubble color indicates the GO P value; bubble size indicates the frequency of the GO term in the Gene Ontology Annotation (UniProt-GOA) Database which annotates all UniProt entries with GO terms. Highly similar GO terms are linked by edges in the graph, where the line width indicates the degree of similarity. (DOCX 179 kb) [file 13073_2017_491_MOESM9_ESM.docx]

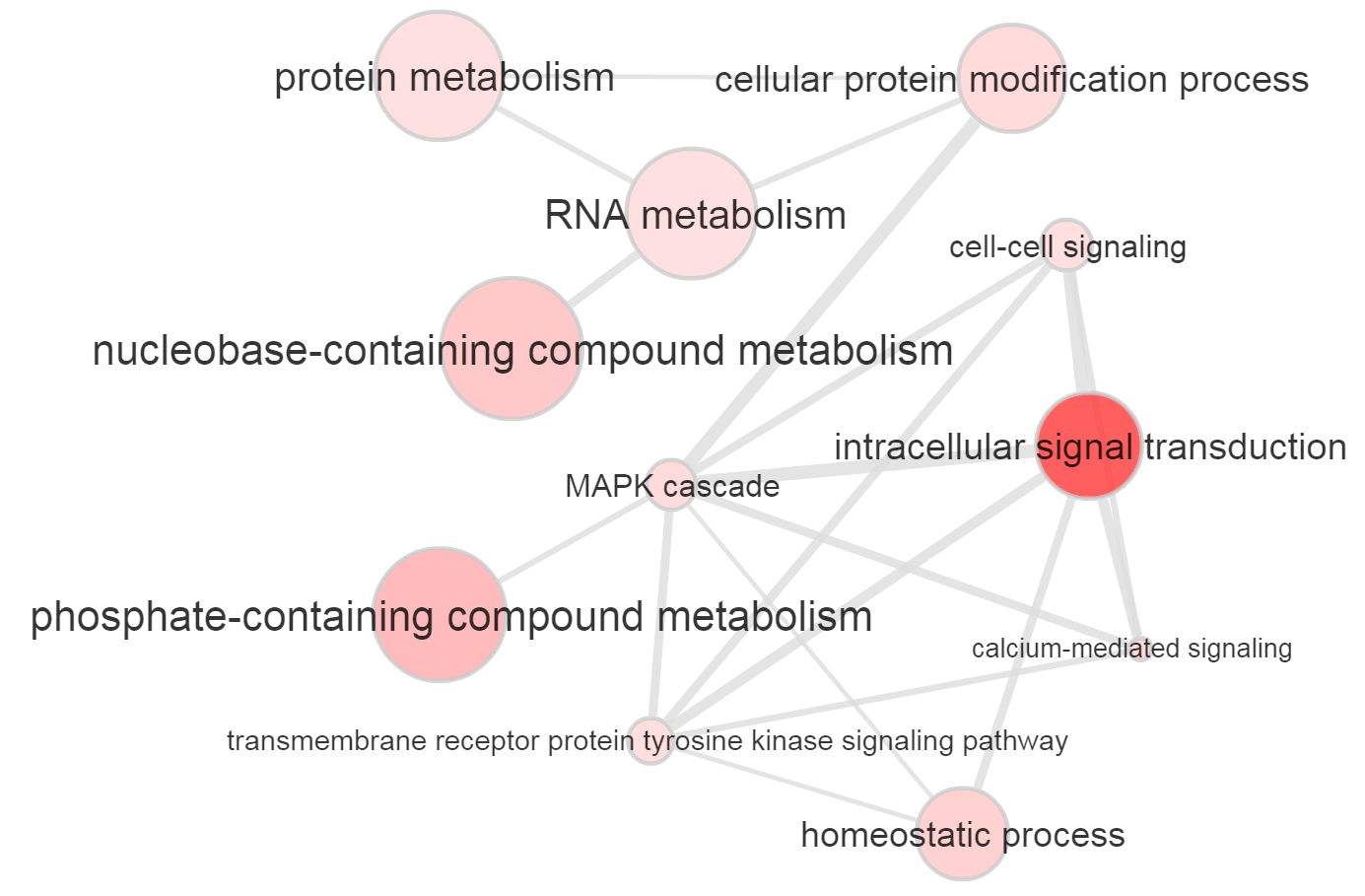


**Additional File 9**: Similarity network based on GO enrichment results related to the AFB-PTSD correlation. Bubble color indicates the GO p-value; bubble size indicates the frequency of the GO term in the Gene Ontology Annotation (UniProt-GOA) Database which annotates all UniProt entries with GO terms. Highly similar GO terms are linked by edges in the graph, where the line width indicates the degree of similarity.

**\**
