# Supplementary material for: A putative causal relationship between genetically determined female body shape and posttraumatic stress disorder
Source: Genome Med. 2017 Nov 27;9:99. doi: 10.1186/s13073-017-0491-4 (PMC5702961; doi:10.1186/s13073-017-0491-4)
Supplement: Supplementary file 10 — SNP-exposure and SNP-outcome coefficients of the variants in included in the WCadj PRS. (DOCX 15 kb) [file 13073_2017_491_MOESM10_ESM.docx]

**Additional File 10:** SNP-exposure and SNP-outcome coefficients of the variants in included in the WC_adj_ PRS.

| **rsID** | **Allele1** | **Allele2** | **PTSD** (outcome) | | **WC_adj_** (exposure1) | | **AFS** (outcome2) | | **NSP** (outcome3) | | |
| --- | --- | --- | --- | --- | --- | --- | --- | --- | --- | --- | --- |
|  |  |  | **Beta** | **SE** | **Beta** | **SE** | **Beta** | **SE** | **Beta** | **SE** |  |
| rs10047100 | C | T | 0.054002 | 0.067 | -0.032 | 0.0052 | -0.0048 | 0.006 | 0.00154 | 0.002 |  |
| rs10195252 | T | C | -0.132 | 0.0591 | 0.035 | 0.0044 | -0.0046 | 0.005 | -0.00058 | 0.002 |  |
| rs10516107 | A | G | -0.0475 | 0.0627 | 0.029 | 0.0046 | -0.0033 | 0.005 | 0.00258 | 0.002 |  |
| rs10923748 | G | C | -0.0964 | 0.0618 | 0.027 | 0.0045 | -0.0007 | 0.005 | 0.00102 | 0.002 |  |
| rs11191295 | A | G | -0.0251 | 0.0589 | -0.029 | 0.0047 | -0.0040 | 0.005 | -0.00403 | 0.002 |  |
| rs12317176 | T | C | -0.13299 | 0.061 | 0.034 | 0.0046 | -0.0031 | 0.005 | -0.00039 | 0.002 |  |
| rs12328675 | T | C | -0.1174 | 0.0852 | 0.039 | 0.0066 | -0.0026 | 0.007 | -0.00091 | 0.002 |  |
| rs12489828 | G | T | 0.086005 | 0.0581 | 0.027 | 0.0044 | -0.0026 | 0.005 | -0.00219 | 0.002 |  |
| rs12549058 | G | T | -0.0315 | 0.1206 | 0.054 | 0.0085 | 0.0039 | 0.010 | 0.00732 | 0.003 |  |
| rs12679556 | G | T | -0.0491 | 0.0672 | 0.035 | 0.0051 | -0.0005 | 0.005 | 0.00233 | 0.002 |  |
| rs1316952 | T | C | -0.1703 | 0.0823 | 0.041 | 0.0064 | -0.0026 | 0.007 | 0.00006 | 0.002 |  |
| rs1358980 | T | C | -0.001 | 0.0599 | 0.053 | 0.0047 | -0.0087 | 0.005 | -0.00276 | 0.002 |  |
| rs1776897 | G | T | -0.1205 | 0.1048 | 0.071 | 0.0087 | -0.0146 | 0.008 | 0.00508 | 0.003 |  |
| rs1884897 | A | G | 0.008999 | 0.06 | 0.035 | 0.0045 | 0.0033 | 0.005 | 0.00053 | 0.002 |  |
| rs1896796 | G | A | 0.102797 | 0.0595 | 0.026 | 0.0044 | -0.0014 | 0.005 | -0.00173 | 0.002 |  |
| rs1936807 | G | C | -0.1132 | 0.0588 | 0.035 | 0.0044 | 0.0021 | 0.005 | -0.00016 | 0.002 |  |
| rs2274432 | A | G | -0.09481 | 0.0619 | 0.027 | 0.0046 | -0.0001 | 0.005 | -0.00040 | 0.002 |  |
| rs2294239 | A | G | -0.036 | 0.0598 | 0.026 | 0.0044 | 0.0006 | 0.005 | -0.00135 | 0.002 |  |
| rs2554380 | T | C | 0.005803 | 0.0723 | 0.031 | 0.0053 | 0.0044 | 0.006 | 0.00185 | 0.002 |  |
| rs2745349 | C | A | -0.103 | 0.0653 | 0.042 | 0.0048 | -0.0017 | 0.005 | -0.00125 | 0.002 |  |
| rs3769891 | A | G | -0.0962 | 0.0659 | 0.031 | 0.0049 | 0.0123 | 0.005 | -0.00427 | 0.002 |  |
| rs6867983 | T | C | 0.050598 | 0.083 | 0.048 | 0.0065 | 0.0040 | 0.007 | 0.00540 | 0.002 |  |
| rs7162542 | G | C | 0.045102 | 0.0598 | 0.037 | 0.0043 | 0.0060 | 0.005 | 0.00116 | 0.002 |  |
| rs7235010 | A | G | -0.0373 | 0.0716 | 0.037 | 0.0053 | -0.0122 | 0.006 | 0.00236 | 0.002 |  |
| rs754133 | A | G | -0.0433 | 0.0622 | 0.029 | 0.0046 | 0.0074 | 0.005 | -0.00373 | 0.002 |  |
| rs7798002 | T | G | 0.075404 | 0.074 | 0.036 | 0.0054 | -0.0177 | 0.006 | -0.00020 | 0.002 |  |
| rs7830933 | A | G | 0.014297 | 0.0682 | 0.033 | 0.0051 | 0.0056 | 0.006 | 0.00045 | 0.002 |  |
| rs849140 | T | C | 0.010801 | 0.0599 | 0.032 | 0.0044 | -0.0060 | 0.005 | -0.00079 | 0.002 |  |
| rs863750 | T | C | -0.1072 | 0.0615 | 0.031 | 0.0044 | -0.0005 | 0.005 | -0.00184 | 0.002 |  |
| rs984222 | C | G | 0.075998 | 0.0605 | -0.036 | 0.0044 | 0.0008 | 0.005 | 0.00004 | 0.002 |  |
| rs9860730 | A | G | 0.032796 | 0.0645 | 0.04 | 0.0048 | -0.0021 | 0.005 | 0.00181 | 0.002 |  |
