# Supplementary material for: A putative causal relationship between genetically determined female body shape and posttraumatic stress disorder
Source: Genome Med. 2017 Nov 27;9:99. doi: 10.1186/s13073-017-0491-4 (PMC5702961; doi:10.1186/s13073-017-0491-4)
Supplement: Supplementary file 11 — SNP-exposure (WCadj associations) and SNP-outcome (PTSD associations) coefficients used in the MR analysis. The lines (see legend) represents the results observed considering different methods. (DOCX 383 kb) [file 13073_2017_491_MOESM11_ESM.docx]

**
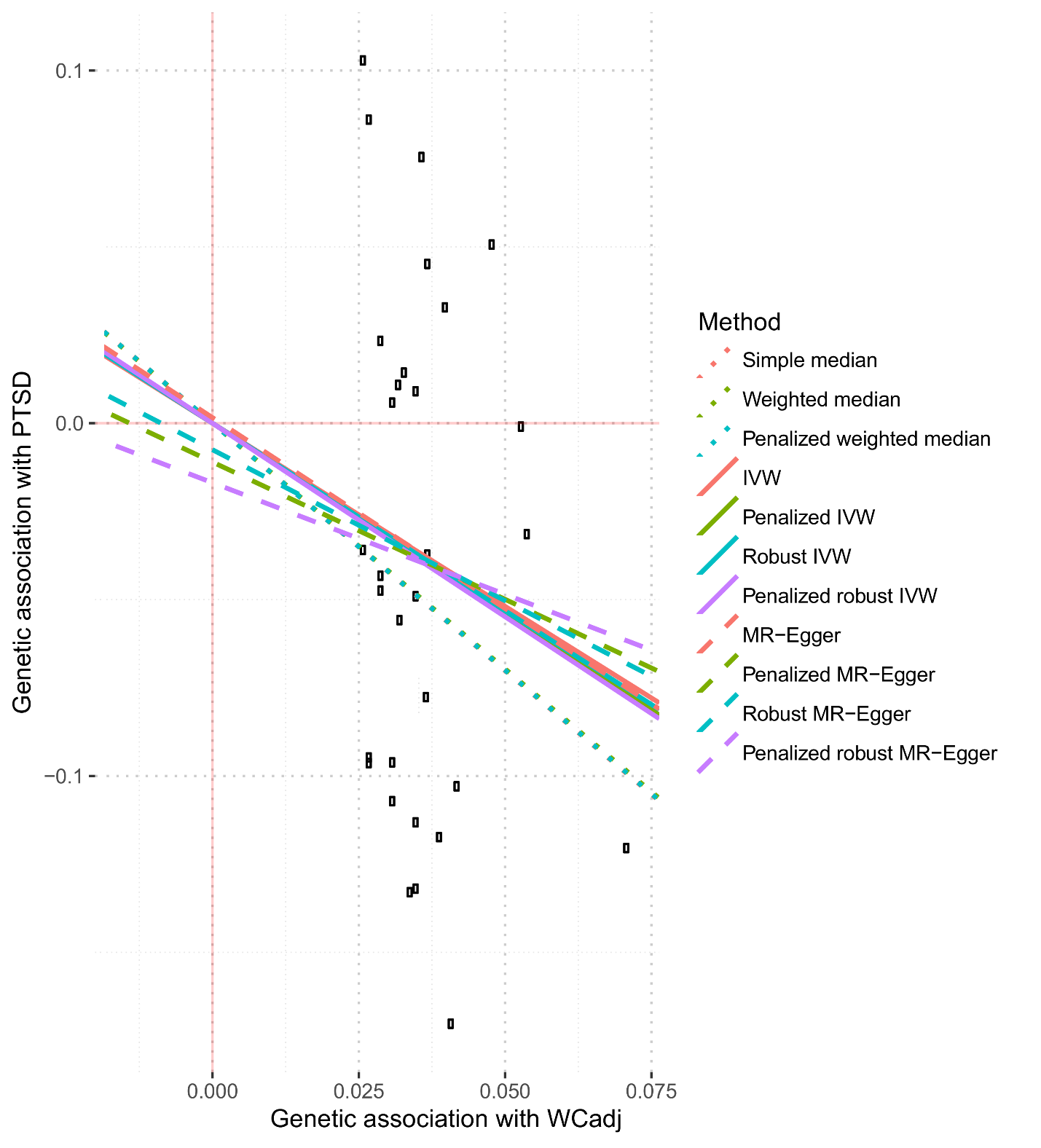
**

**Additional File 11**: SNP-exposure (WCadj associations) and SNP-outcome (PTSD associations) coefficients used in the Mendelian Randomization analysis. The lines (see legend) represents the results observed considering different methods.
