# Supplementary material for: A putative causal relationship between genetically determined female body shape and posttraumatic stress disorder
Source: Genome Med. 2017 Nov 27;9:99. doi: 10.1186/s13073-017-0491-4 (PMC5702961; doi:10.1186/s13073-017-0491-4)
Supplement: Supplementary file 12 — Correlation between PTSD PRS and WCadj. (DOCX 12 kb) [file 13073_2017_491_MOESM12_ESM.docx]

**Additional File 12**: Correlation between PTSD PRS and WC_adj_.

| **PT** | **SNP N** | **R^2^** | **P value** |
| --- | --- | --- | --- |
| 1.00E-06 | 1 | 0.000311 | 0.151 |
| 1.00E-05 | 4 | 0.000137 | 0.246 |
| 1.00E-04 | 35 | 0.000046 | 0.346 |
| 1.00E-03 | 332 | 0.000003 | 0.458 |
| 5.00E-02 | 12826 | 0.000202 | 0.203 |
| 1.00E-01 | 23756 | 0.000411 | 0.118 |
| 3.00E-01 | 60828 | 0.000217 | 0.195 |
| 5.00E-01 | 91947 | 0.000306 | 0.153 |
