# Supplementary material for: A putative causal relationship between genetically determined female body shape and posttraumatic stress disorder
Source: Genome Med. 2017 Nov 27;9:99. doi: 10.1186/s13073-017-0491-4 (PMC5702961; doi:10.1186/s13073-017-0491-4)
Supplement: Supplementary file 13 — Top results of the reverse PRS analysis of PTSD with respect to the main anthropometric traits. (DOCX 12 kb) [file 13073_2017_491_MOESM13_ESM.docx]

**Additional File 13**: Top results of the reverse PRS analysis of PTSD with respect to the main anthropometric traits.

| **Trait** | **PT** | **SNP N** | **R^2^** | **P value** |
| --- | --- | --- | --- | --- |
| BMI | 0.5 | 92340 | 0.00023 | 5.68E-07 |
| WC | 0.5 | 91651 | 0.000192 | 1.26E-05 |
| Height | 1.00E-05 | 4 | 0.000131 | 1.00E-03 |
| WHR | 0.3 | 60751 | 0.000111 | 1.34E-03 |
| HIP | 0.001 | 332 | 0.000113 | 2.83E-03 |
| HIP_adj_ | 0.001 | 332 | 9.05E-05 | 2.83E-03 |
| WHR_adj_ | 0.001 | 332 | 5.1E-05 | 0.026 |
| WC_adj_ | 0.1 | 23756 | 0.000411 | 0.118 |
